# Hibernation and plasma lipids in free-ranging brown bears–implications for diabetes

**DOI:** 10.1371/journal.pone.0291063

**Published:** 2023-09-05

**Authors:** Hasim Tekin, Ole Frøbert, Anne Randi Græsli, Jonas Kindberg, Mesut Bilgin, Karsten Buschard

**Affiliations:** 1 Bartholin Instituttet, Rigshospitalet, Copenhagen, Denmark; 2 Department of Cardiology, Faculty of Health, Örebro University Hospital, Örebro, Sweden; 3 Department of Clinical Medicine, Faculty of Health, Aarhus University, Aarhus, Denmark; 4 Department of Clinical Pharmacology, Aarhus University Hospital, Aarhus, Denmark; 5 Steno Diabetes Center Aarhus, Aarhus University Hospital, Aarhus, Denmark; 6 Department of Forestry and Wildlife Management, Inland Norway University of Applied Sciences, Koppang, Norway; 7 Department of Wildlife, Fish and Environmental Studies, Swedish University of Agricultural Sciences, Umeå, Sweden; 8 Norwegian Institute for Nature Research, Trondheim, Norway; 9 Lipidomics Core Facility, Danish Cancer Institute, Copenhagen, Denmark; University of Illinois, UNITED STATES

## Abstract

Brown bears (*Ursus arctos*) prepare for winter by overeating and increasing adipose stores, before hibernating for up to six months without eating, drinking, and with minimal movement. In spring, the bears exit the den without any damage to organs or physiology. Recent clinical research has shown that specific lipids and lipid profiles are of special interest for diseases such as diabetes type 1 and 2. Furthermore, rodent experiments show that lipids such as sulfatide protects rodents against diabetes. As free-ranging bears experience fat accumulation and month-long physical inactivity without developing diabetes, they could possibly be affected by similar protective measures. In this study, we investigated whether lipid profiles of brown bears are related to protection against hibernation-induced damage. We sampled plasma from 10 free-ranging Scandinavian brown bears during winter hibernation and repeated sampling during active state in the summer period. With quantitative shotgun lipidomics and liquid chromatography-mass spectrometry, we profiled 314 lipid species from 26 lipid classes. A principal component analysis revealed that active and hibernation samples could be distinguished from each other based on their lipid profiles. Six lipid classes were significantly altered when comparing plasma from active state and hibernation: Hexosylceramide, phosphatidylglycerol, and lysophosphatidylglycerol were higher during hibernation, while phosphatidylcholine ether, phosphatidylethanolamine ether, and phosphatidylinositol were lower. Additionally, sulfatide species with shorter chain lengths were lower, while longer chain length sulfatides were higher during hibernation. Lipids that are altered in bears are described by others as relevant for and associated with diabetes, which strengthens their position as potential effectors during hibernation. From this analysis, a range of lipids are suggested as potential protectors of bear physiology, and of potential importance in diabetes.

## Introduction

The brown bear (*Ursus arctos*) has previously been suggested as a model to study type 2 diabetes (T2D), in part due to its dramatic weight gain before the month-long hibernation which is physiologically in contrast to a “sedentary lifestyle” in humans. In spring, bears appear healthy and show no signs of insulin resistance, atherosclerosis, heart failure, or other symptoms observed in humans with long-term sedentary lifestyle [1–3]. In contrast to rodents housed in animal facilities, free-ranging bears do not experience constant stress factors like noise and light.

Lipidome analyses have been used to pair specific lipid profiles to certain diseases and pinpoint lipid species with specific functions [4–6], which could pave the way for lipidomics-based diabetes prediction. Similar to proteins, lipids have important functional roles in cells i.e. by direct interactions with proteins [7]. For example, the sphingolipid sulfatide which aids in maintaining beta cell function and insulin secretion [8] is decreased in the islets of individuals with new-onset type 1 diabetes (T1D) [9]. Furthermore, in newly diagnosed patients with type 1 diabetes, up to one third of the beta cells are inactive [10], which to some extent could be comparable to the state of beta cell activity in hibernating bears. Free fatty acids also specifically target the G-protein coupled receptors named Free Fatty Acid Receptors to activate specific signaling pathways [11]. Several previous studies have analyzed plasma and serum from patients with T1D to use lipid profiles and lipid species as markers of antibody-positive and clinically symptomatic disease states [5, 12–14]. Similar studies have been performed to investigate individuals with prediabetes and T2D [15–17].

Specific lipid species can have specific functional roles both in normal physiology and in animals that hibernate. Lipid classes such as cholesterol and fatty acids can regulate pathways related to torpor, and cholesterol enrichment in the diet of chipmunks improved hibernation parameters whereas saturated fatty acid enrichment worsened them [18]. One study found that feeding of an omega 6-fatty acid-rich diet to alpine marmots increased the activity of myocyte Sarcoendoplasmic Reticulum Calcium ATPase (SERCA) pumps and improved survival through hibernation [19]. Given that bears gain weight and then hibernate without suffering any major physiological deterioration, they could act as a model to detect and describe protective lipid markers and compare these to lipid markers of disease. The purpose of this study was to investigate if bears have alterations in plasma lipid classes or lipid species that could explain how their beta cells remain viable despite months of hibernation-induced inactivity, and thereby avoid developing T1D, T2D, or other diseases.

## Materials and methods

### Sampling of bears

Ten brown bears of 2–3 years of age were sampled in Dalarna, Sweden by a team from the Scandinavian Brown Bear Research Project trained in the capture and handling of free-ranging bears. The bears were sampled between 2012 and 2016 and includes seven female and three male bears. Bears in the project were fitted with neck collars containing GPS and VHF transmitters, which allowed researchers to locate the bears in the dens during their winter hibernation [20], and again in the summer [21] when bears were in their active state. Hibernating bears were anaesthetized with a mixture of medetomidine, zolazepam, tiletamine, and ketamine, and then sampled and examined (S1 Table). At the summer encounter, bears were anaesthetized by darting from a helicopter (with a mixture of medetomidine, zolazepam, and tiletamine), and then sampled and examined; if necessary, ketamine was administered as well. Exact sample dates can be found in the S1 Table; in general, the summer sampling was performed between 98 and 133 days after the first sample, with a mean of 114.7 days between the two sampling events. Venous blood samples were collected into EDTA polypropylene cryotubes, transferred to a field station and centrifuged within 1–2 hours at 2000 g for 7 minutes. Plasma supernatant was collected, frozen at -20°C and kept on dry ice until reaching the laboratory at Örebro University Hospital, Sweden within a maximum of 4 days, where samples were transferred to -80°C storage without breaking the cold chain. The study was approved by the Swedish Ethical Committee on Animal Research (C286/12 and C3/16) and all procedures were performed according to Swedish laws and regulations.

### Lipidomics analysis

Lipid extraction for shotgun lipidomics analysis was performed as reported previously [22]. Lipid extraction was performed with spike of synthetic lipids (S2 Table) and 4 μL bear plasma. The internal standards enabled us to detect 25 lipid classes in five categories. Crude lipid extracts were subjected to FT MS and FT MS/MS analysis on an Orbitrap Fusion Tribrid mass spectrometer (Thermo Fisher Scientific, USA) coupled to a TriVersa NanoMate (Advion Biosciences, USA) which is a direct nanoelectrospray infusion robot; the mass spectrometer was used with optimized settings. The FT MS system operated with R_*m/z* 200_ = 500.000; AGC value of 1×10^5^; maximum injection time of 50 ms; three microscan and FT MS/MS settings with R_*m/z* 200_ = 15.000; AGC value of 2.5×10^4^; maximum injection time of 66 ms; one microscan, while the direct infusion settings were as described previously [22]. Lipid identification and validation was performed with LipidXplorer version 1.2.7 [23] and absolute quantification with homemade software [22].

For sulfatide (SHexCer) extraction, 4 μL of bear plasma in 200 μL ammonium bicarbonate was subjected to a 3-step extraction. Plasma samples were spiked with 10 μL of internal standard SHexCer 30:1;2 (25 pmol), mixed with 1000 μL chloroform/methanol (10:1, v/v), and shaken at 2000 rpm for 10 minutes at 4°C. The lower organic phase was discarded and an additional 1000 μL chloroform/methanol (10:1, v/v) was added, then shaken again as previously. In the final extraction step the lower phase was discarded, 1000 ul chloroform/methanol (2:1, v/v) added, and lastly shaken again as before. The lower phase was transferred to a new tube and vacuum evaporated, then resuspended in 100 μL 6mM ammonium acetate in acetonitrile/methanol (100:2, v/v). Analysis of SHexCer was performed on a (U)HPLC UltiMate 3000 RSLCnano System (Thermo Fisher Scientific) coupled to a Q-Exactive Quadrupole-Orbitrap Mass Spectrometer; a 0.5x150 mm silica column was used (YMC-Pack Silica analytical column with 3 μm particles). LipidXplorer version 1.2.7 was used to identify the SHexCer species and LipidQ used for pmol calculation and determination of time ranges that corresponded to sulfatide species peaks. LC-MS grade solvents were used to compose elution gradients; A: 6 mM ammonium acetate in water; solvent B: 6 mM ammonium acetate in acetonitrile/methanol (100:2, v/v). The gradient profile was: 0–0.1 min, 100–90% B; 0.1–0.5 min, 90% B (isocratic); 0.5–2 min, 90–20% B; 2–6 min, 20% B (isocratic); 6–6.2 min, 20–100% B; 6.2–10 min, 100% B (isocratic). The transfer capillary temperature was 380°C, the spray voltage was 3.8 kV, and FT MS spectra in the negative ionization mode were acquired in the m/z range of 300–950 at the mass resolution of R_m/z 200_ = 35.000; AGC value of 2×105; maximum injection time of 128 ms; three microscans. The measurements for the global shotgun lipidomics were calculated to molar percentage values, from which downstream analyses were performed.

To extract the characteristics of the sulfatide fatty acid chains in the data, we subtracted the sphingoid backbone from the sum composition of SHexCer species with the assumption that the sphingoid backbone corresponds to 18:1:2 (a sphingosine), the most commonly occurring sphingoid backbone of sphingolipids [24, 25]. The SHexCer species were therefore annotated according to this defined assumption: As an example, SHexCer 34:1:2 with its sphingoid backbone (18:1:2) subtracted would thereby leave it with a carbon chain length of 16 and with zero double bonds, thus becoming C16:0 sulfatide. SHexCer measurements were calculated to pmol/μL values, from which the downstream analysis was performed.

### Data analysis and statistics

Heatmaps and PCA plots were generated by using the ClustVis web tool [26] with molar percentages acquired from the shotgun lipidomics assay. This web tool is based on the Principal Component Analysis which takes multivariate data and transforms it to show the variables that affects sample variation the most. The web tool uses R and several R packages such as *ggplot2*, *pheatmap*, and *pcaMethods* to analyze and visualize the data input.

Differences in lipid levels of summer active state and winter hibernation samples were compared by multiple two-tailed paired t-testing in GraphPad Prism version 9.0.0, where the two-stage step-up method of Benjamini, Krieger and Yekutieli was used to correct for multiple comparisons, with a False Discovery Rate (FDR) of 1.0%. Data are shown as means ± standard deviation (SD) in analyses using two-tailed *t* testing. A p-value of ≤0.05 was considered statistically significant. All p-values presented in the main text and figures are corrected p-values. No measurements or outliers measured from the 20 samples were excluded from the analysis. Bar plots and boxplots were generated with GraphPad Prism version 9.0.0. The bubble plot and supplementary figures for chain length and double bonds were generated with R version 4.1.2. All statistics and analyses were made from the global lipidomics molar percentages data, except SHexCer results which were generated from pmol/μL data from the LC-MS assay.

## Results

**Fig 1:**
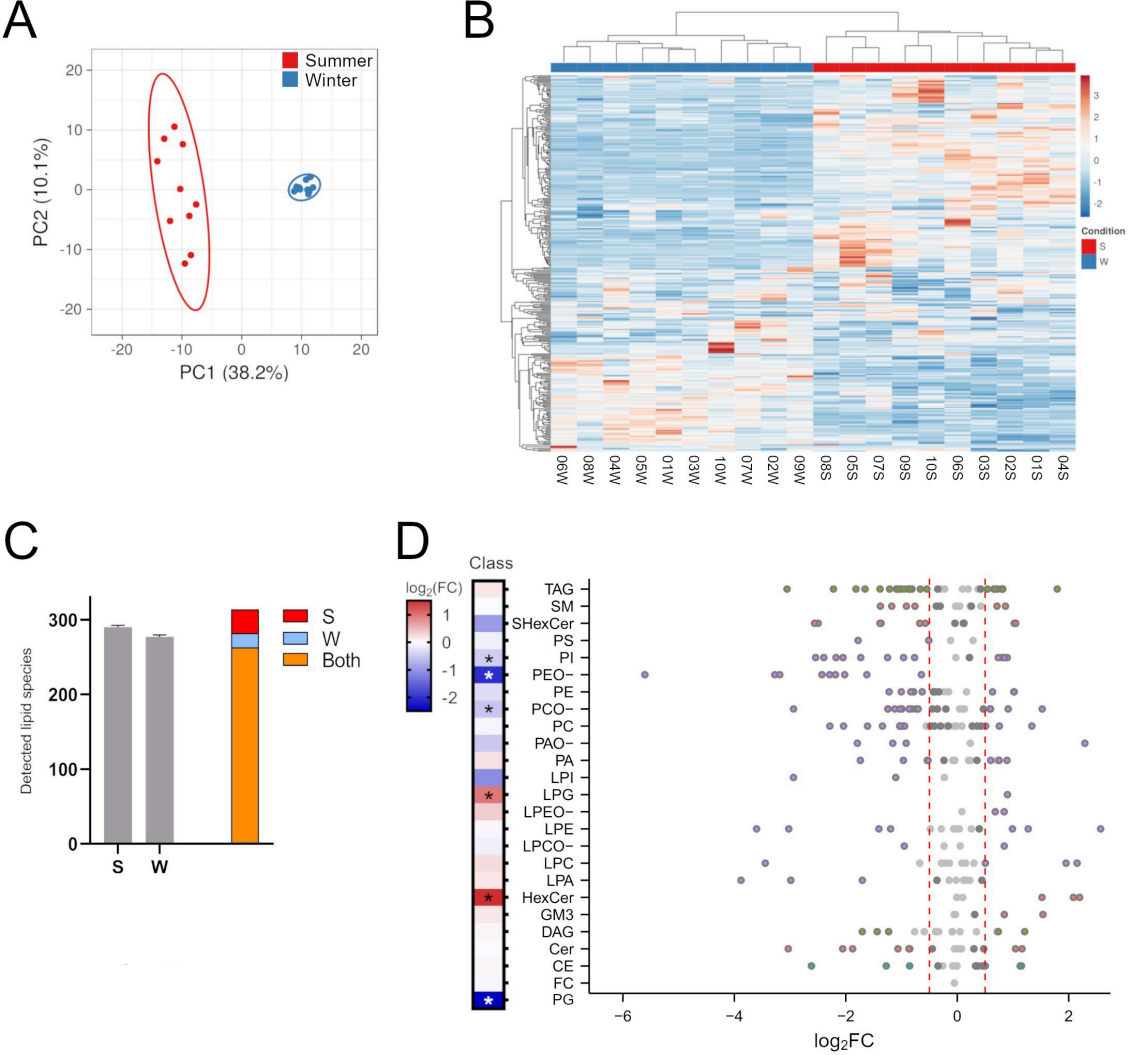
Initial analysis of the lipidomics data set. A. Principal Component Analysis. Red data points are samples from the summer active state (S), blue points from winter hibernation (W). B. Heatmap of the 20 samples in the study. Summer active state (S), winter hibernation state (W). C. Total number of detected lipid species in the summer active state (S), winter hibernation state (W), or both. Error bars shows SD. D. Heatmap and bubble plot of log_2_ fold changes (log_2_FC) for lipid species in each respective class. In each class in the bubble plot, the lipid species with a significant log_2_FC are colored, whereas nonsignificant species remain gray (adjusted p-value threshold of 0.05). Lipid species with a mean level of 0 in the summer active state is not shown in the plot since their log_2_FC could not be calculated. The lower limit for log_2_FC in the heatmap is similarly set to -2.5, as the few species with an extraordinary high fold change would skew plotting of the remaining data. Significantly altered classes are marked with a single asterisk (p<0.05, paired t-test with correction for multiple testing). All classes in the bubble plot were calculated from molar percentages acquired via shotgun metabolomics, except for SHexCer which was calculated from pmol/μL values acquired with a more sensitive LC-MS approach. The red vertical lines in the bubble plot mark log_2_FC thresholds of 0.5 and -0.5. The class “FC” denotes free cholesterol, refer to the methods for abbreviations for the other classes. n = 20 for all figures.

The characteristics of the ten bears sampled in the study is shown in S1 Table. An initial plotting of all measured lipids as a Principal Component Analysis plot (Fig 1A) and heatmap (Fig 1B) revealed that the 20 samples mainly clustered according to the sample collection time of summer active state or winter hibernation i.e. all samples in the hibernation cluster had the same levels of the same lipids (Fig 1B). Sex- and age differences did not affect the clustering either, again highlighting the similarity of lipid profiles during each of the two sampling seasons. In total, we detected an average of 291 lipid species in the summer active samples and 278 in winter hibernation samples (Fig 1C). 32 species were detected in summer active samples only, 19 in winter hibernation samples only, and 263 species detected in both sample types.

**Fig 2:**
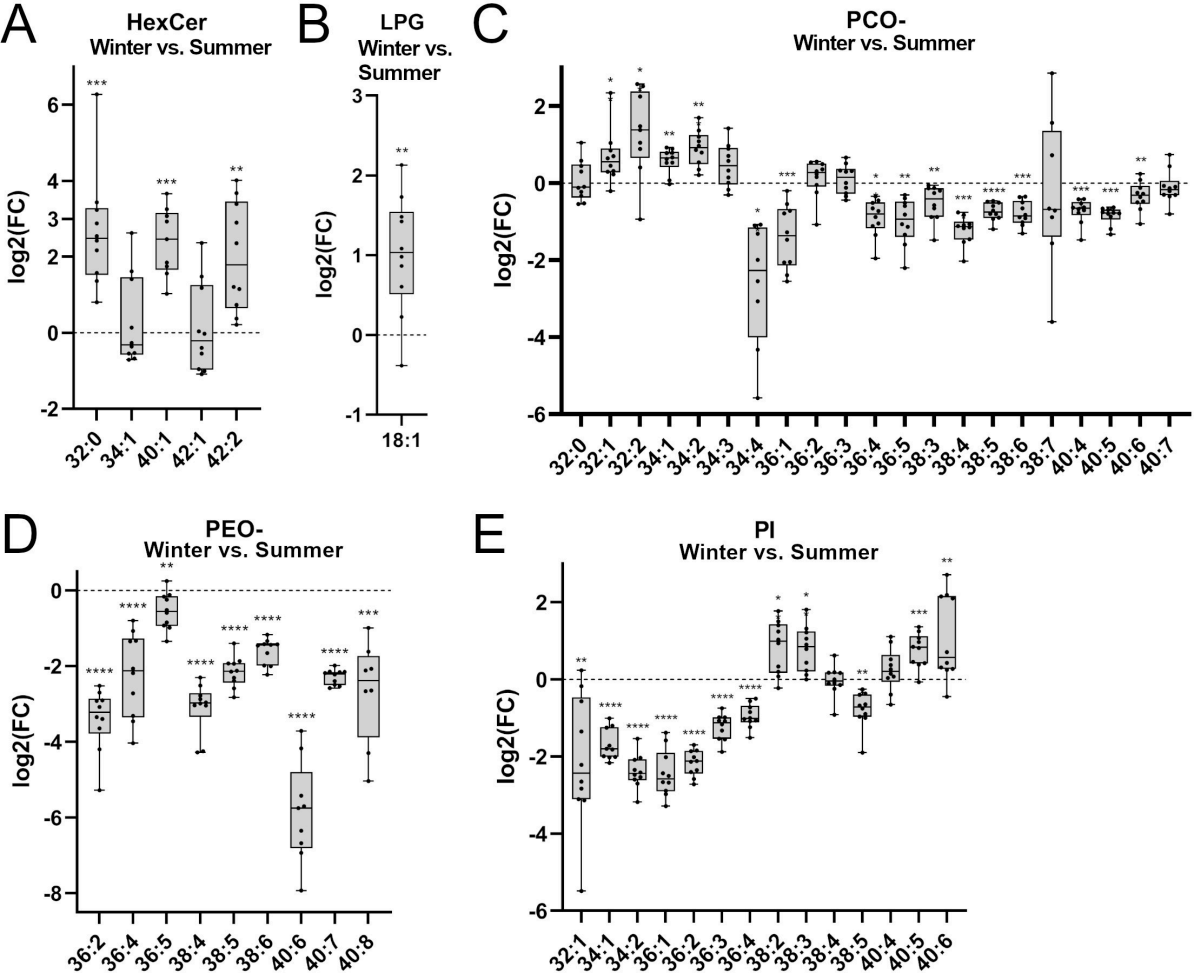
Species-level differences calculated as fold changes for the significantly altered classes. Shotgun lipidomics was used to measure differences in paired samples, and log_2_ fold change (log_2_FC) was calculated for winter divided by summer (W/S). The box shows the median value and 25^th^ and 75^th^ percentiles, while whiskers show the min and max values. All plots show mean molar percentage of lipids measured in plasma. Data are shown as means from a paired t-test, and error bars indicate ± SD, n = 20. A. Hexosylceramide. B. lysophosphatidylglycerol. C. phosphatidylcholine ether. D. phosphatidylethanolamine ether. E. phosphatidylinositol. *p < 0.05, **p < 0.01, ***p < 0.001, ****p < 0.0001. All p-values are corrected for multiple testing as described in the methods.

Class-level analysis of the samples with shotgun lipidomics (Fig 1D, left panel) revealed that total levels of three lipid classes were significantly higher during hibernation: hexosylceramide (HexCer, p = 0.002), phosphatidylglycerol (PG, p < 0.001), and lysophosphatidylglycerol (LPG, p = 0.005). We could only quantify PG in winter hibernation samples, as no PG was present in any of the summer active state samples. Additionally, three classes in the glycerophospholipid category were significantly lower during hibernation: phosphatidylcholine ether (PCO-, p = 0.002), phosphatidylethanolamine ether (PEO-, p < 0.001), and phosphatidylinositol (PI, p = 0.005). The remaining lipid classes were unaltered on class-level but had changes in individual lipid species (S1 Fig). It was noted that the lysophosphatidylserine (LPS) measured (Fig 2D, left panel) was only detected in hibernation samples, but that the variation was too high be statistically significant. Since HexCer, PG, and LPG classes were higher in winter hibernation samples, it is possible that these have protective roles during hibernation.

To evaluate substantial changes in all the measured lipid species, we stratified the log_2_FC with the previously calculated p-values to create a bubble plot (Fig 1D, right panel). In this plot, all lipid species were grouped into classes and arranged by their log_2_FC, and lipid species that differed significantly between hibernation and active state were colored. The log_2_FC of 51 of the 314 species could not be calculated due to a mean lipid level of 0 in either the active or hibernation state, and these species are therefore not shown. Of these species, 233 were significantly altered during hibernation with 113 species being lower and 120 higher. The total level of each lipid class was determined by the sum of the levels for each lipid species within that class, and therefore the difference between hibernation and active state levels were determined by adding up all changes for all lipid species. For the significantly changed classes described previously, nearly all species changed similarly, i.e. all PEO- species were lower during hibernation and therefore the total level of PEO- was also lower. Contrary to this, classes such as sphingomyelin (SM), phosphatidylcholine (PC), and cholesteryl ester (CE) had some lipid species that were higher during hibernation and some that were lower, which in total resulted in nonsignificant changes in the respective lipid class level.

After analyzing class-wide changes, we evaluated lipid changes at the species-level of the lipid classes that were significantly altered. We calculated and visualized log_2_ fold changes for all species; species with a mean value of 0 in either of the conditions could not yield a usable log_2_FC and could therefore not be visualized. The lipid classes HexCer, LPG, and PG were higher during hibernation. For HexCer, five species were higher during hibernation, and three species were unchanged (Fig 2A, five species shown). For LPG, the two measured species were higher during hibernation (Fig 2B, one species shown). For PG, all three measured species were higher during hibernation as well (none could be visualized). The lipid classes PEO-, PCO-, and PI were lower during hibernation, which could indicate a protective role in regard to preventing diabetes during this inactive period. Of the 26 PCO- species measured, 16 species were lower during hibernation, four were higher, and six were unchanged (Fig 2C, 21 species shown). All species that were higher during hibernation had lower chain lengths as well. For PEO-, all 11 measured PEO- species were lower during hibernation (Fig 2D, 9 species shown). Of the 20 PI species measured, 13 species were higher during hibernation, four lower, and three unchanged (Fig 2E, 14 species shown). Two of the three species with higher levels had a chain length of 38 and either one or two double bonds, and the third had a chain length of 40 and six double bonds.

**Fig 3:**
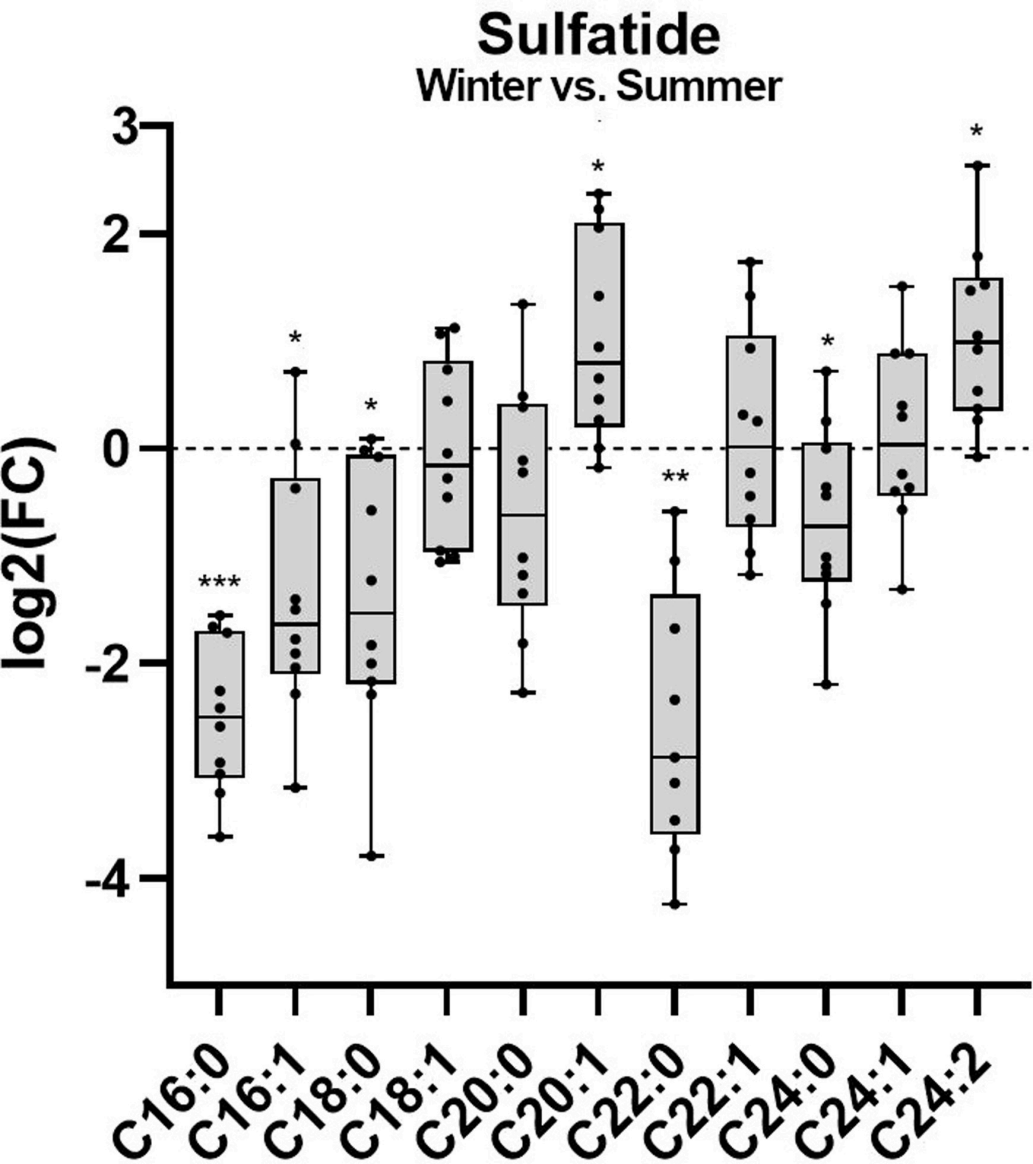
Species-level differences calculated as fold changes for sulfatide. An optimized LC-MS approach was used to measure and acquire SHexCer pmol/μL data. SHexCer species were then converted to sulfatide species by subtracting the sphingosine (18:1;2) backbone, as described in the methods, and log_2_ fold changes (log2FC) calculated for W/S. The box shows the median value and 25^th^ and 75^th^ percentiles, while whiskers show the min and max values. All plots show mean molar percentage of lipids measured in plasma. Data are shown as means from a paired t-test, and error bars indicate ± SD, n = 20. *p < 0.05, **p < 0.01, ***p < 0.001, ****p < 0.0001. All p-values are corrected for multiple testing as described in the methods.

The LC-MS analysis of sulfatide by measurement of sulfatide (SHexCer, Fig 3) revealed that of the eleven species measured, the levels of five lipid species were significantly lower, two significantly higher, and four unaltered, when comparing samples from hibernation with samples from active state. The sulfatides with the shortest chain lengths of 16 carbons (C16:0 and C16:1) and one sulfatide with 18 carbons (C18:0) were lower during hibernation as shown by a lower log_2_FC for W/S (C16:0 p < 0.001, C16:1 p = 0.012, C18:0 p = 0.012). Sulfatides C18:1 and C20:0 were unaltered (C18:1 p = 0.70, C20:0 p = 0.14), whereas C20:1 was higher during hibernation (p = 0.012). Unsaturated sulfatides C22:1 and C24:1 were also unaltered during hibernation, whereas C24:0 was lower and C24:2 higher (C22:1 p = 0.70, C24:1 p = 0.70, C24:0 p = 0.050, C24:2 p = 0.010). It should be noted that C16 and C24 sulfatides made up the largest molar percentage of the samples relative to the other sulfatides measured (shown in S2 Fig panel B).

Grouping the lipids by chain length (S3 Fig) revealed that levels of shorter length lipids were higher during hibernation, and the longer lengths were lower for lipid classes diacylglycerol (DAG), triacylglycerol (TAG), phosphatidic acid (PA), phosphatidic acid ether (PAO-), and phosphatidylcholine ether (PCO-). SM followed a reverse trend, as the shorter lengths of 32 and 34 were lower and the longer chain lengths from 36–42 were higher during hibernation. For LPE, the middle chain lengths were higher during hibernation and the shortest and longest chain lengths were simultaneously lower during hibernation. Chain lengths were higher during hibernation for five out of six measured HexCer species. The lysophosphatidylcholine (LPC) class also had an increase in all chain lengths during hibernation. When grouping on the number of double bonds (dbs) instead (S4 Fig), Cer lipids with 0 and 1 dbs were lower in hibernation while lipids with 2 and 3 dbs were higher. DAG followed a reverse trend where lipids with 0, 1, or 2 dbs were higher during hibernation, and lipids with 3, 4, or 5 dbs were lower during hibernation; PA, PAO- and TAG had similar changes. For PCO-, lipids with 0, 4, 5, 6, and 7 dbs were lower during hibernation. All dbs of HexCer were higher in hibernation, all dbs of PEO- and LPI were lower, and lipids with all dbs except dbs 6 were lower for PE during hibernation.

## Discussion

### Summary and main findings

This study used plasma samples from summer active state and winter hibernating brown bears to examine differences in their lipidomic profiles and pinpoint class- or species-level changes with implications for T1D and T2D. Other studies have previously performed metabolic [27–29] and lipidomic-specific analyses [30] in hibernating bears but have measured a significantly smaller set of lipid classes and species than presented here. Furthermore, bears have also been studied to gain knowledge on other diseases, such as venous thromboembolism in humans [31]. Our shotgun lipidomics analysis revealed that six lipid classes were significantly altered in the two seasons: PG, LPG, and HexCer were higher during hibernation, whereas PCO-, PEO-, and PI were lower. Furthermore, sulfatides measured with LC-MS were also altered, as short chain length sulfatides were lower during hibernation, while long-chain sulfatides were unaltered or higher.

### Translational implications of lipids altered during hibernation

In our analysis, C16 sulfatides were lower during hibernation, while the three C24 sulfatides were each altered differently; C24:0 was lower, C24:1 unaltered, and C24:2 higher during hibernation. We have previously shown that fenofibrate injection into NOD mice, which spontaneously develop T1D, specifically increased C24 sulfatides and other long-chain sphingolipids, and that these animals had lower T1D incidence when compared to control animals [32]. Furthermore, injections of sulfatide C24:0 decreased T1D incidence in NOD mice [33]. C16 sulfatides regulate insulin activity and production, while C24 sulfatides are implicated in suppression of immune activity [33, 34]. The higher level of C24 sulfatides during hibernation could therefore indicate presence of a more anti-inflammatory environment in the bears.

The lipid class PCO- is found to be altered in both T1D and T2D [13, 14, 35]. A cohort of children who later developed T1D had decreased levels of total PCO- in cord-blood samples obtained before their clinical debut [13]. Furthermore, PCO- decreases were observed in a similar adolescent T1D study as well [36]. Specific PCO- species were also altered in the peripheral blood mononuclear cells of children with T1D compared to non-diabetic children [14]. In children with T1D, PCO- 36:5 were higher, and PCO- 38:4 and 38:5 lower. Furthermore, PCO- was associated with T2D in plasma from adults with T2D compared to healthy adults [35]. In bears, the level of total PCO- was significantly lower during hibernation, as were the levels of the mentioned PCO- species. As the relation between PCO- and diabetes is unclear and indicates both a protective and damaging role, determining whether the PCO- present in the bears has a functional role in protecting the bear during hibernation should be investigated in future studies.

The lipid class LPG, which was higher in bears during hibernation, has been described by a single study in relation to diabetes. An untargered metabolomics study on serum from healthy controls and patients with T2D sampled six years after clinical diagnosis found that LPG predicted T2D in individuals with baseline HbA1c levels. In essence, these low-risk individuals would normally be diagnosed as healthy based on their Hb1Ac in a clinical setting. Specifcally, LPG 12:0 is suggested as a potent marker to diagnose such individuals at an early state [37].

The lipid class PI makes up 10–20% of all phospholipids and normally functions as cellular messengers and regulators of cellular mechanisms. Downstream enzymes convert PI into PIP2 and PIP3 that have a range of functions, including insulin secretion for PIP2 [38], and the role of PI is therefore in part examined by investigating PIP2, PIP3, and relevant enzymes. In beta cells, PIP2 elevation decreased K^ATP^-channel sensitivity and impaired glucose-stimulated insulin secretion [39]. In adipocyte cultures, blocking PIP3 metabolism induced insulin resistance [40], and PI was associated with T2D in a muscle biopsy analysis from patients with T2D [41]. Thus, it is possible that the lower PI in bears during hibernation is part of a mechanism that induces insulin resistance in order to ration the sparse glucose stores. To investigate this further, it would be relevant to measure the levels of PIP2, PIP3, and enzymes of PI biosynthesis in bears.

HexCer levels were also higher during hibernation. A 11 year long follow-up study of healthy adults found a positive association between plasma HexCer and characteristics for obesity and T2D such as body-mass index, HbA1c, and insulin resistance [42]. In a cohort for metabolic syndrome, a condition which comprises obesity and insulin resistance, HexCer was inverserly associated with metabolic syndrome markers [43]. The literature for the role of HexCer in diabetes is limited, and thus more information is needed in order to document whether HexCer is a protective lipid in bear hibernation.

PG is reported to inhibit TLR-mediated inflammation in primary mouse keratinocytes and RAW264.7 macrophages [44, 45], and lung surfactant which contains PC and PG inhibited LPS-induced inflammation in macrophages [46]. Furthermore, total PG and PI, as well as PG species 34:1, 34:2, 36:1, and 36:2, were associated with T2D in a human T2D cohort [35]. Presence of inflammation is especially relevant for diabetes as low-grade inflammation is a condition associated with T2D in patients [47], and white adipose tissue releases high levels of proinflammatory cytokines [48], which could cause inflammation in bears with large adipose stores prior to hibernation. Since PG levels in bears were higher during hibernation, this could indicate that bears transition towards an anti-inflammatory state in order to protect from the effects of hibernation i.e. sedentary lifestyle and adipose tissue accumulation.

Another lipid with relevance to inflammation is LPS, which was present in hibernating state plasma but absent in active state plasma. LPS is a common component of cell walls of Gram-negative bacteria, and its presence induces an acute inflammatory response with release of proinflammatory cytokines [49]. Its presence in hibernation samples therefore indicates that the hibernating bear is attacked by incoming bacteria. A previous proteomics-metabolomics study detected an upregulation of antimicrobial defense proteins [50], which in theory could be upregulated to protect against an increased risk of bacterial infection during hibernation. In contrast, the same study detected suppression of innate and acquired immune defenses, which appears counterintuitive since antimicrobial defenses were upregulated. Further studies into this would elucidate the interaction between these two protective systems.

### Translational implications of lipids not altered during hibernation

Some of the lipid classes that were not altered during hibernation are described as implicated in diabetes. One of these is DAG which is known to induce insulin resistance associated with T2D and obesity [51]. Intracellular DAG levels are regulated by diacylglycerol kinases (DGKs) whose inhibition causes DAG accumulation and reduced insulin secretion, and DGKδ deficiency is found in patients with T2D [52]. As DGKs are activated by glucose stimulation in beta cells, the euglycemia reported in a previous bear study [29] could indicate that bears are protected from diabetic complications by maintaining a healthy DAG metabolism despite weight gain and hibernation. Similarly, TAG which was unaltered in the bears is also a marker of T2D. Individuals with high TAG levels are up to 54% more likely to develop T2D [53], whereas children that progress to T1D has decreased levels of triglycerides when compared to serum from their clinical debut. A lipolysis study in bears found that that inhibitors of lipolysis were upregulated in the adipose tissue during active state, which is thought to increase insulin sensitivity and promote weight gain [54]. The lack of change in TAG or DAG levels could be a marker of resilience towards developing diabetes or other complications during hibernation, which should be investigated further.

Contrary to DAG and TAG, the LPC class is described as protective when elevated and dangerous when decreased in relation to diabetes. Plasma LPC levels were decreased in high fat diet-fed mice relative to non-obese controls, and in a small cohort of humans with obesity and T2D [55]. Furthermore, stimulating streptozotocin-injected mice and obese db/db mice with LPC lowered blood glucose levels [56]. However, some studies have described the reverse i.e. that LPC is protective at decreased levels. LPC is independently associated with diabetes [57], and hepatic arylsulfatase A increased LPC and LPA in lipid rafts, which improved muscle insulin sensitivity [58]. In a previous study we found that the ratio of pro- and anti-inflammatory LPC lipids were altered in mice with T1D [32]: NOD mice given a fenofibrate-rich diet which protected against spontaneous T1D also had a lower inflammatory LPC ratio (pro-inflammatory LPC / anti-inflammatory LPC) compared to the control diet group. Thus, this LPC ratio could in part serve as a risk marker for T1D development. For bears, this ratio remained similar during hibernation and the active period (S5 Fig). Since the bears had no change in LPC levels nor ratio, the question remains whether the given level of LPC or certain LPC species present in total has pro- or anti-inflammatory effects, and if these affect mechanisms during hibernation that prevent disease formation.

## Limitations and conclusion

This study has potential limitations. Bears are inevitably stressed by our approach in their dens in winter and by helicopter darting in summer, which could have altered some plasma lipids. The fact that we only have two samples instead of serial samples from each bear is also a limitation, since we only get a brief observational window to detect any changes. Furthermore, as the lipidomics analysis is highly sensitive, even minute sources on contamination would result in a positive signal, such as lipids on the skin surface where blood is sampled from. Future studies should take these factors into account both during sampling and subsequent analysis to account for the consequences of the limitations, and determine lipid profiles in the autumn season prior to hibernation to investigate why the bears can become temporarily obese without developing metabolic syndrome.

With this study, we determined that the lipidome of brown bears was altered during hibernation, manifested as changes in the total levels of PG, PI, PCO-, PEO-, HexCer, LPG, and sulfatides. Furthermore, lipid classes such as GM3 and DAG that are altered in patients with diabetes were not altered in bears. These classes have different roles in disease promotion and prevention, whose implication in hibernation should be elucidated further. From this data, we propose that C24 sulfatides have the potential for further investigation, as they are significantly altered in the bears and described in literature to protect against diabetes and other diseases. Other lipid classes such as PI that were altered during hibernation are also relevant to study further, as are lipids such as DAG and TAG that are altered in patients with diabetes but unaltered in bears. Combining lipidomics studies with animal- and bioassays would confirm the causal relevance of lipid classes and species in mechanisms controlling the lack of disease during hibernation, with the goal of applying these findings translationally.

## Supporting information

S1 Table Characteristics of sampled bears. Each bear has a unique WXXXX number followed by its capture and sampling season (“S” for summer active state and “W” for winter hibernation) and two numbers denoting the year; “W1303 S14” is thus a sampling of bear W1303 in the summer of 2014.
(XLSX)

S2 Table Internal standard mixture for shotgun lipidomics. (XLSX)

S1 Fig Measured molar percentages for all detected species. (PDF)

S2 Fig Total levels of all detected classes, calculated by adding all species in a respective class together. All values are in molar percentages, except sulfatide which is in pmol/μL. A. Classes detected by shotgun lipidomics. Glycerophospholipids (GPL) sphingolipids (SL), lysoglycerophospholipids (LGPL), glycerolipids (GL), sterols (ST). Refer to the methods section for lipid class abbreviations. B. Sulfatide. Refer to the methods section for info on sulfatide measurements.
(PDF)

S3 Fig Lipid species grouped by classes and number of carbons in its fatty acid chain (length, x axes). The y axes are shown in molar percentages (lipid quantities). The bars show the mean value in each of the two conditions “S” (summer active state) and “W” (winter hibernation).
(PDF)

S4 Fig Lipid species grouped by classes and number of double bonds (db, x axes). The y axes are shown in molar percentages (lipid quantities). The bars show the mean value in each of the two conditions “S” (summer active state) and “W” (winter hibernation).
(PDF)

S5 Fig Ratio of pro- and anti-inflammatory LPC lipids in samples. Pro-inflammatory lipids are classified as lipids with 0 or 1 double bonds, whereas anti-inflammatory lipids have 2–6 double bonds. A lower ratio indicates higher levels of anti-inflammatory LPCs and thus a lower proportion of inflammation. The ratio was calculated separately for each bear sample, and all samples were then pooled together in the two conditions. “S” is summer active state samples, “W” winter hibernation samples. A paired *t* test showed that no significant difference existed between the groups (*p* = 0.46).
(PDF)

S1 Dataset Shotgun lipidomics dataset, LC-MS dataset, and test statistics. (CSV)

## References

[1] Fröbert O, Frøbert AM, Kindberg J, Arnemo JM, Overgaard MT. The brown bear as a translational model for sedentary lifestyle-related diseases. Journal of Internal Medicine. 2020;287(3):263–70. doi: 10.1111/joim.12983 31595572

[2] Chazarin B, Storey KB, Ziemianin A, Chanon S, Plumel M, Chery I, et al. Metabolic reprogramming involving glycolysis in the hibernating brown bear skeletal muscle. Frontiers in Zoology. 2019 May 6;16(1):12. doi: 10.1186/s12983-019-0312-2 31080489 PMC6503430

[3] Rigano KS, Gehring JL, Evans Hutzenbiler BD, Chen AV, Nelson OL, Vella CA, et al. Life in the fat lane: seasonal regulation of insulin sensitivity, food intake, and adipose biology in brown bears. Journal of comparative physiology B, Biochemical, systemic, and environmental physiology. 2017;187(4):649–76. doi: 10.1007/s00360-016-1050-9 27987017

[4] Al-Sari N, Schmidt S, Suvitaival T, Kim M, Trošt K, Ranjan AG, et al. Changes in the lipidome in type 1 diabetes following low carbohydrate diet: Post-hoc analysis of a randomized crossover trial. Endocrinology, Diabetes & Metabolism. 2021;4(2):e00213. doi: 10.1002/edm2.213 33855215 PMC8029500

[5] Overgaard AJ, Weir JM, Jayawardana K, Mortensen HB, Pociot F, Meikle PJ. Plasma lipid species at type 1 diabetes onset predict residual beta-cell function after 6 months. Metabolomics. 2018;14(12):158. doi: 10.1007/s11306-018-1456-3 30830451 PMC6280838

[6] Rhee EP, Cheng S, Larson MG, Walford GA, Lewis GD, McCabe E, et al. Lipid profiling identifies a triacylglycerol signature of insulin resistance and improves diabetes prediction in humans. J Clin Invest. 2011 Apr 1;121(4):1402–11. doi: 10.1172/JCI44442 21403394 PMC3069773

[7] Sych T, Levental KR, Sezgin E. Lipid-Protein Interactions in Plasma Membrane Organization and Function. Annu Rev Biophys. 2022 May 9;51:135–56. doi: 10.1146/annurev-biophys-090721-072718 34982570 PMC12101515

[8] Buschard K, Hoy M, Bokvist K, Olsen HL, Madsbad S, Fredman P, et al. Sulfatide Controls Insulin Secretion by Modulation of ATP-sensitive K+-Channel Activity and Ca2+-Dependent Exocytosis in Rat Pancreatic -Cells. Diabetes. 2002 Aug 1;51(8):2514–21. 12145165 10.2337/diabetes.51.8.2514

[9] Holm LJ, Krogvold L, Hasselby JP, Kaur S, Claessens LA, Russell MA, et al. Abnormal islet sphingolipid metabolism in type 1 diabetes. Diabetologia. 2018 Jul;61(7):1650–61. doi: 10.1007/s00125-018-4614-2 29671030 PMC6445476

[10] Krogvold L, Wiberg A, Edwin B, Buanes T, Jahnsen FL, Hanssen KF, et al. Insulitis and characterisation of infiltrating T cells in surgical pancreatic tail resections from patients at onset of type 1 diabetes. Diabetologia. 2016 Mar 1;59(3):492–501. doi: 10.1007/s00125-015-3820-4 26602422

[11] Secor JD, Fligor SC, Tsikis ST, Yu LJ, Puder M. Free Fatty Acid Receptors as Mediators and Therapeutic Targets in Liver Disease. Front Physiol. 2021 Apr 7;12:656441. doi: 10.3389/fphys.2021.656441 33897464 PMC8058363

[12] Lamichhane S, Ahonen L, Sparholt Dyrlund T, Dickens AM, Siljander H, Hyöty H, et al. Cord-Blood Lipidome in Progression to Islet Autoimmunity and Type 1 Diabetes. Biomolecules. 2019 Jan;9(1):33. doi: 10.3390/biom9010033 30669674 PMC6359525

[13] Oresic M, Simell S, Sysi-Aho M, Näntö-Salonen K, Seppänen-Laakso T, Parikka V, et al. Dysregulation of lipid and amino acid metabolism precedes islet autoimmunity in children who later progress to type 1 diabetes. J Exp Med. 2008 Dec 22;205(13):2975–84. doi: 10.1084/jem.20081800 19075291 PMC2605239

[14] Sen P, Dickens AM, López-Bascón MA, Lindeman T, Kemppainen E, Lamichhane S, et al. Metabolic alterations in immune cells associate with progression to type 1 diabetes. Diabetologia. 2020;63(5):1017–31. doi: 10.1007/s00125-020-05107-6 32043185 PMC7145788

[15] Fretts AM, Jensen PN, Hoofnagle A, McKnight B, Howard BV, Umans J, et al. Plasma Ceramide Species Are Associated with Diabetes Risk in Participants of the Strong Heart Study. J Nutr. 2020 May;150(5):1214–22. doi: 10.1093/jn/nxz259 31665380 PMC7198314

[16] Liu J, Bai L, Wang W, Song Y, Zhao W, Li Q, et al. LC-MS-Based Lipidomic Analysis of Serum Samples from Patients with Type 2 Diabetes Mellitus (T2DM). Dis Markers. 2022 Feb 12;2022:5559470. doi: 10.1155/2022/5559470 35190756 PMC8858047

[17] Lu J, Lam SM, Wan Q, Shi L, Huo Y, Chen L, et al. High-Coverage Targeted Lipidomics Reveals Novel Serum Lipid Predictors and Lipid Pathway Dysregulation Antecedent to Type 2 Diabetes Onset in Normoglycemic Chinese Adults. Diabetes Care. 2019 Aug 27;42(11):2117–26. doi: 10.2337/dc19-0100 31455687

[18] Kolomiytseva IK. Lipids in mammalian hibernation and artificial hypobiosis. Biochemistry (Mosc). 2011 Dec;76(12):1291–9. doi: 10.1134/S0006297911120029 22150274

[19] Ruf T, Arnold W. Effects of polyunsaturated fatty acids on hibernation and torpor: a review and hypothesis. American Journal of Physiology-Regulatory, Integrative and Comparative Physiology. 2008 Mar;294(3):R1044–52. doi: 10.1152/ajpregu.00688.2007 18171691

[20] Evans AL, Sahlén V, Støen OG, Fahlman Å, Brunberg S, Madslien K, et al. Capture, Anesthesia, and Disturbance of Free-Ranging Brown Bears (Ursus arctos) during Hibernation. PLOS ONE. 2012 Jul 16;7(7):e40520. doi: 10.1371/journal.pone.0040520 22815757 PMC3398017

[21] Arnemo J, Evans A. Biomedical Protocols for Free-ranging Brown Bears, Wolves, Wolverines and Lynx. 2017 Mar.

[22] Nielsen IØ, Olsen AV, Dicroce-Giacobini J, Papaleo E, Andersen KK, Jäättelä M, et al. Comprehensive Evaluation of a Quantitative Shotgun Lipidomics Platform for Mammalian Sample Analysis on a High-Resolution Mass Spectrometer. Journal of the American Society for Mass Spectrometry [Internet]. 2020 Feb 18 [cited 2022 Nov 4]; Available from: doi: 10.1021/jasms.9b00136 32129994

[23] Herzog R, Schuhmann K, Schwudke D, Sampaio JL, Bornstein SR, Schroeder M, et al. LipidXplorer: A Software for Consensual Cross-Platform Lipidomics. PLOS ONE. 2012 Jan 17;7(1):e29851. doi: 10.1371/journal.pone.0029851 22272252 PMC3260173

[24] Menaldino DS, Bushnev A, Sun A, Liotta DC, Symolon H, Desai K, et al. Sphingoid bases and de novo ceramide synthesis: enzymes involved, pharmacology and mechanisms of action. Pharmacological Research. 2003 May 1;47(5):373–81. doi: 10.1016/s1043-6618(03)00054-9 12676511

[25] Chen Y, Liu Y, Sullards MC, Merrill AH. An Introduction to Sphingolipid Metabolism and Analysis by New Technologies. Neuromolecular Med. 2010;12(4):306–19. doi: 10.1007/s12017-010-8132-8 20680704 PMC2982954

[26] Metsalu T, Vilo J. ClustVis: a web tool for visualizing clustering of multivariate data using Principal Component Analysis and heatmap. Nucleic Acids Research. 2015 Jul 1;43(W1):W566–70. doi: 10.1093/nar/gkv468 25969447 PMC4489295

[27] Shimozuru M, Nagashima A, Tanaka J, Tsubota T. Seasonal changes in the expression of energy metabolism-related genes in white adipose tissue and skeletal muscle in female Japanese black bears. Comparative Biochemistry and Physiology Part B: Biochemistry and Molecular Biology. 2016 Jun 1;196–197:38–47. doi: 10.1016/j.cbpb.2016.02.001 26880364

[28] Sommer F, Ståhlman M, Ilkayeva O, Arnemo JM, Kindberg J, Josefsson J, et al. The Gut Microbiota Modulates Energy Metabolism in the Hibernating Brown Bear Ursus arctos. Cell Reports. 2016 Feb 23;14(7):1655–61. doi: 10.1016/j.celrep.2016.01.026 26854221

[29] Stenvinkel P, Fröbert O, Anderstam B, Palm F, Eriksson M, Bragfors-Helin AC, et al. Metabolic Changes in Summer Active and Anuric Hibernating Free-Ranging Brown Bears (Ursus arctos). PLoS One. 2013 Sep 9;8(9):e72934. doi: 10.1371/journal.pone.0072934 24039826 PMC3767665

[30] Giroud S, Chery I, Bertile F, Bertrand-Michel J, Tascher G, Gauquelin-Koch G, et al. Lipidomics Reveals Seasonal Shifts in a Large-Bodied Hibernator, the Brown Bear. Front Physiol. 2019 Apr 12;10:389. doi: 10.3389/fphys.2019.00389 31031634 PMC6474398

[31] Thienel M, Müller-Reif JB, Zhang Z, Ehreiser V, Huth J, Shchurovska K, et al. Immobility-associated thromboprotection is conserved across mammalian species from bear to human. Science. 2023 Apr 14;380(6641):178–87. doi: 10.1126/science.abo5044 37053338

[32] Holm LJ, Haupt-Jorgensen M, Giacobini JD, Hasselby JP, Bilgin M, Buschard K. Fenofibrate increases very-long-chain sphingolipids and improves blood glucose homeostasis in NOD mice. Diabetologia. 2019 Dec;62(12):2262–72. doi: 10.1007/s00125-019-04973-z 31410530 PMC6861358

[33] Subramanian L, Blumenfeld H, Tohn R, Ly D, Aguilera C, Maricic I, et al. NKT cells stimulated by long fatty acyl chain sulfatides significantly reduce the incidence of type 1 diabetes in nonobese diabetic mice [corrected]. PLoS One. 2012;7(5):e37771. 10.1371/journal.pone.0037771 PMC3359325 22649557

[34] The C24:0 Sulfatide Isoform as an Important Molecule in Type 1 Diabetes ‐ PubMed [Internet]. [cited 2023 Jan 31]. Available from: https://pubmed.ncbi.nlm.nih.gov/36624946/10.31083/j.fbl2712331 36624946

[35] Meikle PJ, Wong G, Barlow CK, Weir JM, Greeve MA, MacIntosh GL, et al. Plasma Lipid Profiling Shows Similar Associations with Prediabetes and Type 2 Diabetes. PLOS ONE. 2013 Sep 27;8(9):e74341. doi: 10.1371/journal.pone.0074341 24086336 PMC3785490

[36] La Torre D, Seppänen-Laakso T, Larsson HE, Hyötyläinen T, Ivarsson SA, Lernmark Å, et al. Decreased Cord-Blood Phospholipids in Young Age–at–Onset Type 1 Diabetes. Diabetes. 2013 Nov;62(11):3951–6. doi: 10.2337/db13-0215 23929934 PMC3806611

[37] Lu Y, Wang Y, Ong CN, Subramaniam T, Choi HW, Yuan JM, et al. Metabolic signatures and risk of type 2 diabetes in a Chinese population: an untargeted metabolomics study using both LC-MS and GC-MS. Diabetologia. 2016 Nov 1;59(11):2349–59. doi: 10.1007/s00125-016-4069-2 27514531

[38] Chang W, Hatch GM, Wang Y, Yu F, Wang M. The relationship between phospholipids and insulin resistance: From clinical to experimental studies. Journal of Cellular and Molecular Medicine. 2019;23(2):702–10. doi: 10.1111/jcmm.13984 30402908 PMC6349352

[39] Lin CW, Yan F, Shimamura S, Barg S, Shyng SL. Membrane phosphoinositides control insulin secretion through their effects on ATP-sensitive K+ channel activity. Diabetes. 2005 Oct;54(10):2852–8. doi: 10.2337/diabetes.54.10.2852 16186385 PMC1350465

[40] Chen G, Raman P, Bhonagiri P, Strawbridge AB, Pattar GR, Elmendorf JS. Protective Effect of Phosphatidylinositol 4,5-Bisphosphate against Cortical Filamentous Actin Loss and Insulin Resistance Induced by Sustained Exposure of 3T3-L1 Adipocytes to Insulin. J Biol Chem. 2004 Sep 17;279(38):39705–9. doi: 10.1074/jbc.C400171200 15277534 PMC2413414

[41] Beeson M, Sajan MP, Dizon M, Grebenev D, Gomez-Daspet J, Miura A, et al. Activation of Protein Kinase C-ζ by Insulin and Phosphatidylinositol-3,4,5-(PO4)3 Is Defective in Muscle in Type 2 Diabetes and Impaired Glucose Tolerance: Amelioration by Rosiglitazone and Exercise. Diabetes. 2003 Aug 1;52(8):1926–34. 12882907 10.2337/diabetes.52.8.1926

[42] Chew WS, Torta F, Ji S, Choi H, Begum H, Sim X, et al. Large-scale lipidomics identifies associations between plasma sphingolipids and T2DM incidence. JCI Insight. 2019 Jun 4;5(13):e126925, 126925. doi: 10.1172/jci.insight.126925 31162145 PMC6629294

[43] Berkowitz L, Salazar C, Ryff CD, Coe CL, Rigotti A. Serum sphingolipid profiling as a novel biomarker for metabolic syndrome characterization. Front Cardiovasc Med. 2022;9:1092331. doi: 10.3389/fcvm.2022.1092331 36578837 PMC9791223

[44] Choudhary V, Uaratanawong R, Patel RR, Patel H, Bao W, Hartney B, et al. Phosphatidylglycerol inhibits toll-like receptor-mediated inflammation by danger-associated molecular patterns. J Invest Dermatol. 2019 Apr;139(4):868–77. doi: 10.1016/j.jid.2018.10.021 30391260 PMC7309510

[45] Chen WW, Chao YJ, Chang WH, Chan JF, Hsu YHH. Phosphatidylglycerol Incorporates into Cardiolipin to Improve Mitochondrial Activity and Inhibits Inflammation. Sci Rep. 2018 Mar 20;8:4919. doi: 10.1038/s41598-018-23190-z 29559686 PMC5861085

[46] Kuronuma K, Mitsuzawa H, Takeda K, Nishitani C, Chan ED, Kuroki Y, et al. Anionic Pulmonary Surfactant Phospholipids Inhibit Inflammatory Responses from Alveolar Macrophages and U937 Cells by Binding the Lipopolysaccharide-interacting Proteins CD14 and MD-2. J Biol Chem. 2009 Sep 18;284(38):25488–500. doi: 10.1074/jbc.M109.040832 19584052 PMC2757950

[47] Okdahl T, Wegeberg AM, Pociot F, Brock B, Størling J, Brock C. Low-grade inflammation in type 2 diabetes: a cross-sectional study from a Danish diabetes outpatient clinic. BMJ Open. 2022 Dec 14;12(12):e062188. doi: 10.1136/bmjopen-2022-062188 36517105 PMC9756179

[48] Park YM, Myers M, Vieira-Potter VJ. Adipose Tissue Inflammation and Metabolic Dysfunction: Role of Exercise. Mo Med. 2014;111(1):65–72. doi: 10.1146/annurev-physiol-021909-135846 24645302 PMC6179510

[49] Ngkelo A, Meja K, Yeadon M, Adcock I, Kirkham PA. LPS induced inflammatory responses in human peripheral blood mononuclear cells is mediated through NOX4 and Giα dependent PI-3kinase signalling. Journal of Inflammation. 2012 Jan 12;9(1):1. 22239975 10.1186/1476-9255-9-1 PMC3293082

[50] Welinder KG, Hansen R, Overgaard MT, Brohus M, Sønderkær M, von Bergen M, et al. Biochemical Foundations of Health and Energy Conservation in Hibernating Free-ranging Subadult Brown Bear Ursus arctos. J Biol Chem. 2016 Oct 21;291(43):22509–23. doi: 10.1074/jbc.M116.742916 27609515 PMC5077189

[51] Erion DM, Shulman GI. Diacylglycerol-mediated insulin resistance. Nat Med. 2010 Apr;16(4):400–2. doi: 10.1038/nm0410-400 20376053 PMC3730126

[52] Kaneko YK, Ishikawa T. Diacylglycerol Signaling Pathway in Pancreatic β-Cells: An Essential Role of Diacylglycerol Kinase in the Regulation of Insulin Secretion. Biological and Pharmaceutical Bulletin. 2015;38(5):669–73. 25947912 10.1248/bpb.b15-00060

[53] Zhao J, Zhang Y, Wei F, Song J, Cao Z, Chen C, et al. Triglyceride is an independent predictor of type 2 diabetes among middle-aged and older adults: a prospective study with 8-year follow-ups in two cohorts. Journal of Translational Medicine. 2019 Dec 3;17(1):403. doi: 10.1186/s12967-019-02156-3 31801571 PMC6894231

[54] Jessen N, Nielsen TS, Vendelbo MH, Viggers R, Støen O, Evans A, et al. Pronounced expression of the lipolytic inhibitor G0/G1 Switch Gene 2 (G0S2) in adipose tissue from brown bears (Ursus arctos) prior to hibernation. Physiol Rep. 2016 Apr 25;4(8):e12781. doi: 10.14814/phy2.12781 27117803 PMC4848729

[55] Barber MN, Risis S, Yang C, Meikle PJ, Staples M, Febbraio MA, et al. Plasma Lysophosphatidylcholine Levels Are Reduced in Obesity and Type 2 Diabetes. PLoS One. 2012 Jul 25;7(7):e41456. 22848500 10.1371/journal.pone.0041456 PMC3405068

[56] Yea K, Kim J, Yoon JH, Kwon T, Kim JH, Lee BD, et al. Lysophosphatidylcholine Activates Adipocyte Glucose Uptake and Lowers Blood Glucose Levels in Murine Models of Diabetes. J Biol Chem. 2009 Dec 4;284(49):33833–40. doi: 10.1074/jbc.M109.024869 19815546 PMC2797153

[57] Burlakova EB, Karagezyan KG, Amirkhanyan OM, Ovakimyan SS, Sekoyan ES. Disorders of tissue transformations of lysophosphatidylcholines at experimental pancreatic diabetes in white rats and peculiarities of the corrective effect of low-energy laser radiation of an extremely low intensity. Doklady Biochemistry and Biophysics. 2010 Aug;433(1):145–7. doi: 10.1134/S1607672910040010 20714843

[58] Montgomery MK, Bayliss J, Nie S, De Nardo W, Keenan SN, Miotto PM, et al. Deep proteomic profiling unveils arylsulfatase A as a non-alcoholic steatohepatitis inducible hepatokine and regulator of glycemic control. Nat Commun. 2022 Mar 10;13:1259. doi: 10.1038/s41467-022-28889-2 35273160 PMC8913628

