# Supplementary material for: Hibernation and plasma lipids in free-ranging brown bears–implications for diabetes
Source: PLoS One. 2023 Sep 5;18(9):e0291063. doi: 10.1371/journal.pone.0291063 (PMC10479895; doi:10.1371/journal.pone.0291063)
Supplement: S1 Fig — (PDF) [file pone.0291063.s003.pdf]

S3

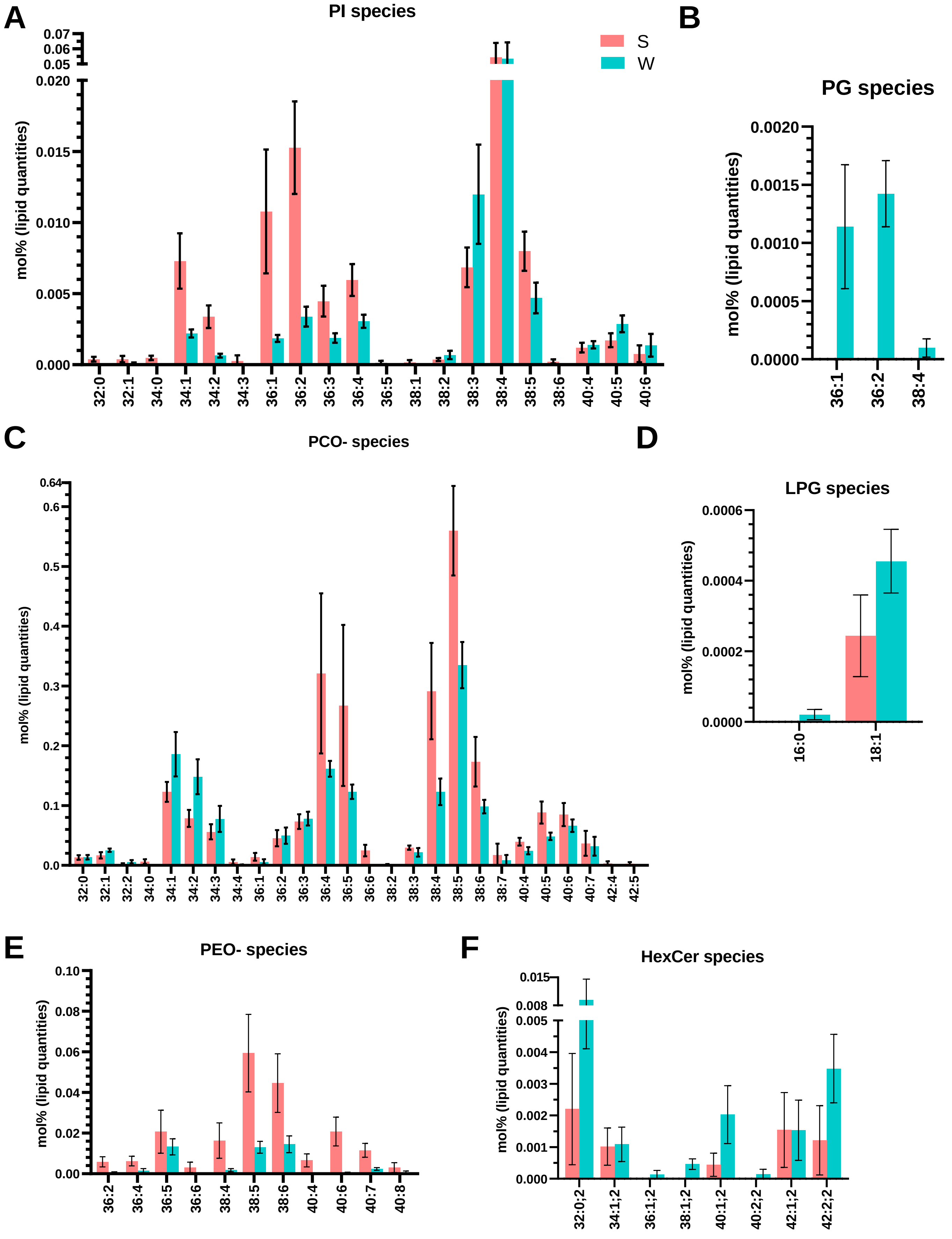

A
PI species
S
W
mol% (lipid quantities)
0.07
0.06
0.05
0.020
0.015
0.010
0.005
0.000
32:0
32:1
34:0
34:1
34:2
34:3
36:1
36:2
36:3
36:4
36:5
38:1
38:2
38:3
38:4
38:5
38:6
40:4
40:5
40:6
B
PG species
mol% (lipid quantities)
0.0020
0.0015
0.0010
0.0005
0.0000
36:1
36:2
38:4
C
PCO- species
mol% (lipid quantities)
0.64
0.6
0.5
0.4
0.3
0.2
0.1
0.0
32:0
32:1
32:2
34:0
34:1
34:2
34:3
34:4
36:1
36:2
36:3
36:4
36:5
36:6
38:2
38:3
38:4
38:5
38:6
38:7
40:4
40:5
40:6
40:7
42:4
42:5
D
LPG species
mol% (lipid quantities)
0.0006
0.0004
0.0002
0.0000
16:0
18:1
E
PEO- species
mol% (lipid quantities)
0.10
0.08
0.06
0.04
0.02
0.00
36:2
36:4
36:5
36:6
38:4
38:5
38:6
40:4
40:6
40:7
40:8
F
HexCer species
mol% (lipid quantities)
0.015
0.008
0.005
0.004
0.003
0.002
0.001
0.000
32:0;2
34:1;2
36:1;2
38:1;2
40:1;2
40:2;2
42:1;2
42:2;2

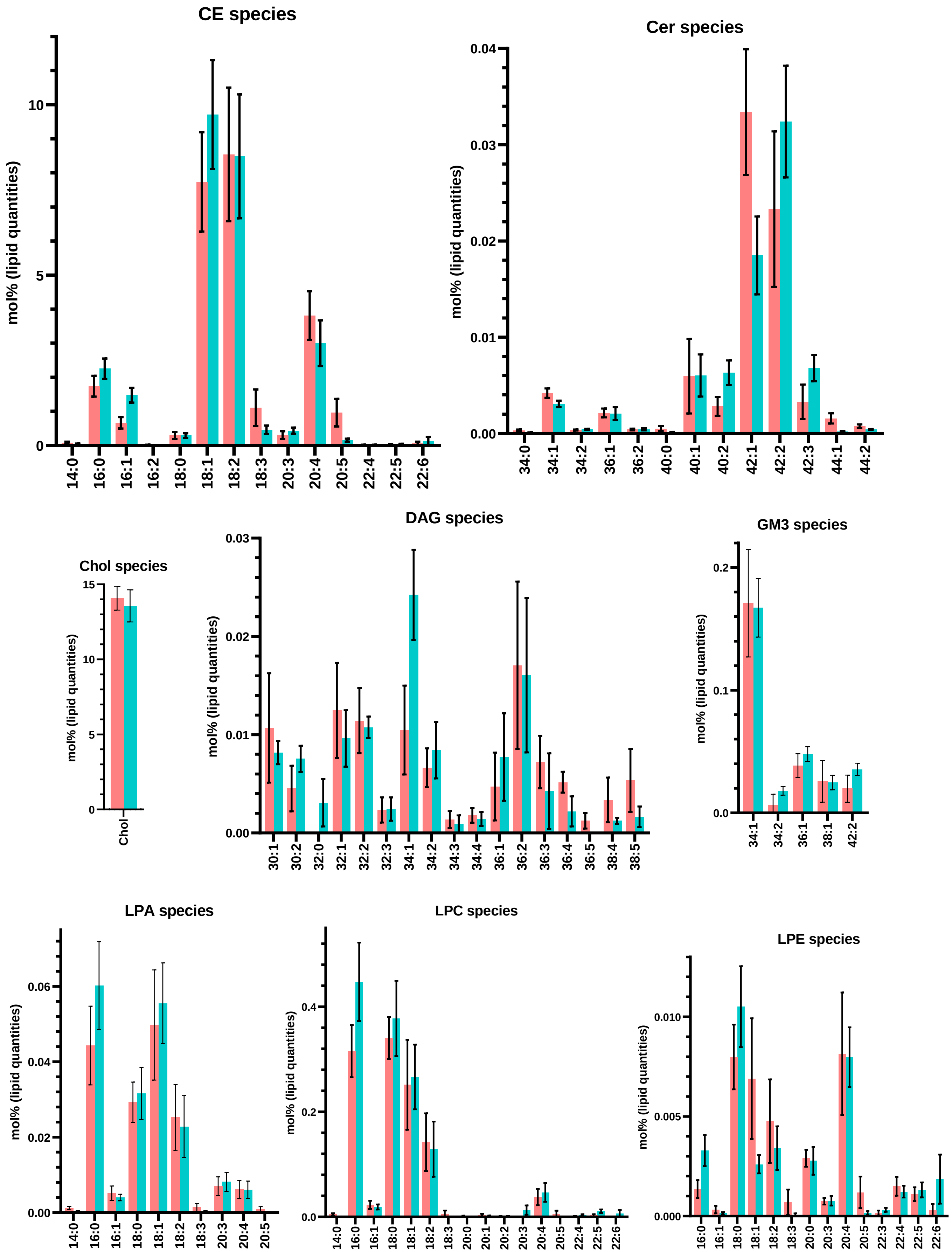
CE species
mol% (lipid quantities)
0
5
10
14:0
16:0
16:1
16:2
18:0
18:1
18:2
18:3
20:3
20:4
20:5
22:4
22:5
22:6
Cer species
mol% (lipid quantities)
0.00
0.01
0.02
0.03
0.04
34:0
34:1
34:2
36:1
36:2
40:0
40:1
40:2
42:1
42:2
42:3
44:1
44:2
Chol species
mol% (lipid quantities)
0
5
10
15
Chol
DAG species
mol% (lipid quantities)
0.00
0.01
0.02
0.03
30:1
30:2
32:0
32:1
32:2
32:3
34:1
34:2
34:3
34:4
36:1
36:2
36:3
36:4
36:5
38:4
38:5
GM3 species
mol% (lipid quantities)
0.0
0.1
0.2
34:1
34:2
36:1
38:1
42:2
LPA species
mol% (lipid quantities)
0.00
0.02
0.04
0.06
14:0
16:0
16:1
18:0
18:1
18:2
18:3
20:3
20:4
20:5
LPC species
mol% (lipid quantities)
0.0
0.2
0.4
14:0
16:0
16:1
18:0
18:1
18:2
18:3
20:0
20:1
20:2
20:3
20:4
20:5
22:4
22:5
22:6
LPE species
mol% (lipid quantities)
0.000
0.005
0.010
16:0
16:1
18:0
18:1
18:2
18:3
20:0
20:3
20:4
20:5
22:3
22:4
22:5
22:6

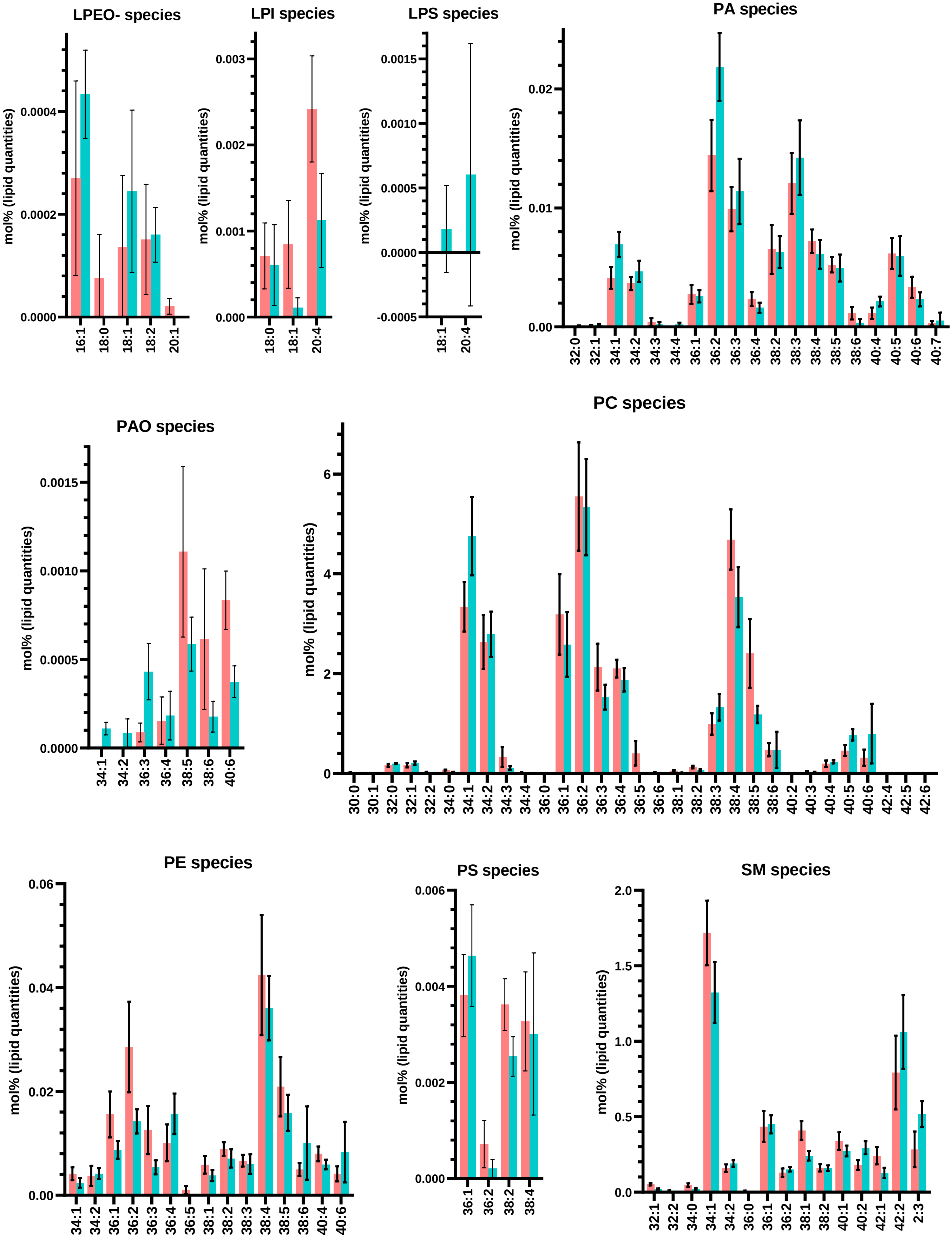

LPEO- species
mol% (lipid quantities)
0.0000
0.0002
0.0004
16:1
18:0
18:1
18:2
20:1
LPI species
mol% (lipid quantities)
0.000
0.001
0.002
0.003
18:0
18:1
20:4
LPS species
mol% (lipid quantities)
-0.0005
0.0000
0.0005
0.0010
0.0015
18:1
20:4
PA species
mol% (lipid quantities)
0.00
0.01
0.02
32:0
32:1
34:1
34:2
34:3
34:4
36:1
36:2
36:3
36:4
38:2
38:3
38:4
38:5
38:6
40:4
40:5
40:6
40:7
PAO species
mol% (lipid quantities)
0.0000
0.0005
0.0010
0.0015
34:1
34:2
36:3
36:4
38:5
38:6
40:6
PC species
mol% (lipid quantities)
0
2
4
6
30:0
30:1
32:0
32:1
32:2
34:0
34:1
34:2
34:3
34:4
36:0
36:1
36:2
36:3
36:4
36:5
36:6
38:1
38:2
38:3
38:4
38:5
38:6
40:2
40:3
40:4
40:5
40:6
42:4
42:5
42:6
PE species
mol% (lipid quantities)
0.00
0.02
0.04
0.06
34:1
34:2
36:1
36:2
36:3
36:4
36:5
38:1
38:2
38:3
38:4
38:5
38:6
40:4
40:6
PS species
mol% (lipid quantities)
0.000
0.002
0.004
0.006
36:1
36:2
38:2
38:4
SM species
mol% (lipid quantities)
0.0
0.5
1.0
1.5
2.0
32:1
32:2
34:0
34:1
34:2
36:0
36:1
36:2
38:1
38:2
40:1
40:2
42:1
42:2
2:3

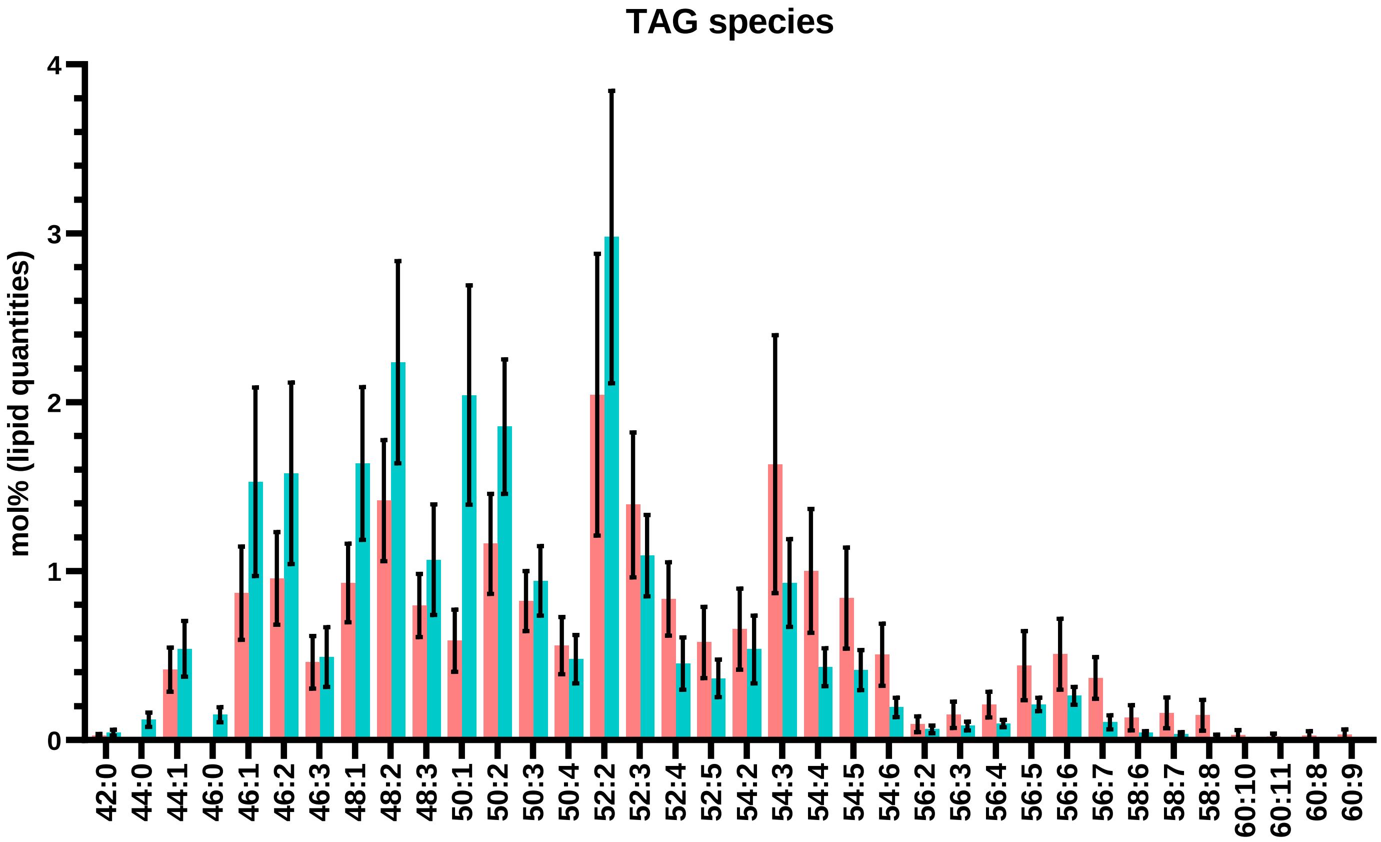

TAG species
mol% (lipid quantities)
4
3
2
1
0
42:0
44:0
44:1
46:0
46:1
46:2
46:3
48:1
48:2
48:3
50:1
50:2
50:3
50:4
52:2
52:3
52:4
52:5
54:2
54:3
54:4
54:5
54:6
56:2
56:3
56:4
56:5
56:6
56:7
58:6
58:7
58:8
60:10
60:11
60:8
60:9
