# Supplementary figures and images for: Hibernation and plasma lipids in free-ranging brown bears–implications for diabetes
Source: PLoS One. 2023 Sep 5;18(9):e0291063. doi: 10.1371/journal.pone.0291063 (PMC10479895; doi:10.1371/journal.pone.0291063)

A

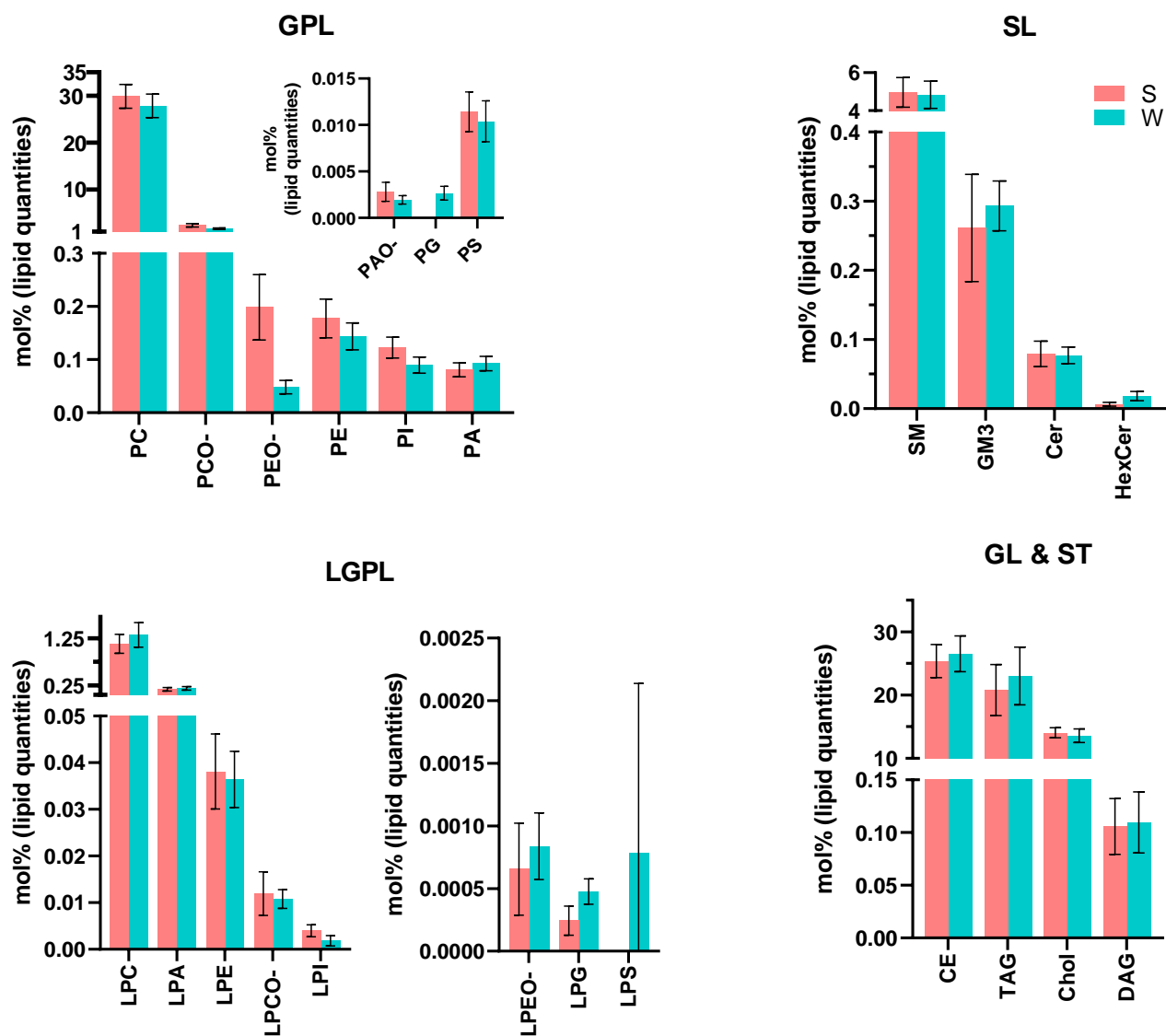

B

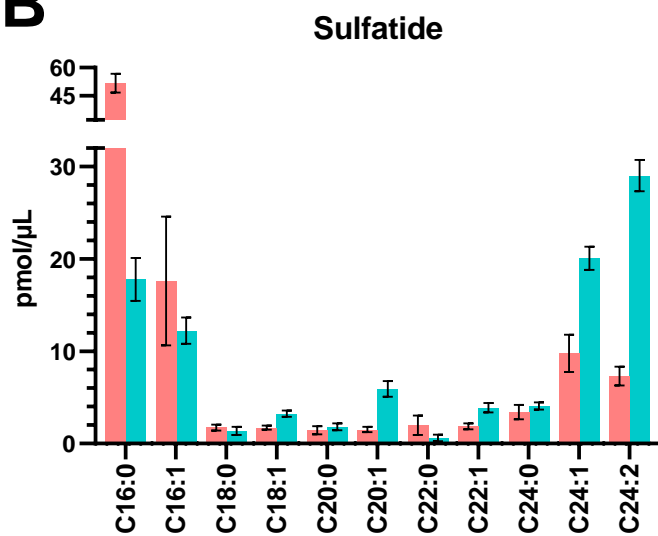

Supplement: S2 Fig — All values are in molar percentages, except sulfatide which is in pmol/μL. A. Classes detected by shotgun lipidomics. Glycerophospholipids (GPL) sphingolipids (SL), lysoglycerophospholipids (LGPL), glycerolipids (GL), sterols (ST). Refer to the methods section for lipid class abbreviations. B. Sulfatide. Refer to the methods section for info on sulfatide measurements. (PDF) [file pone.0291063.s004.pdf]

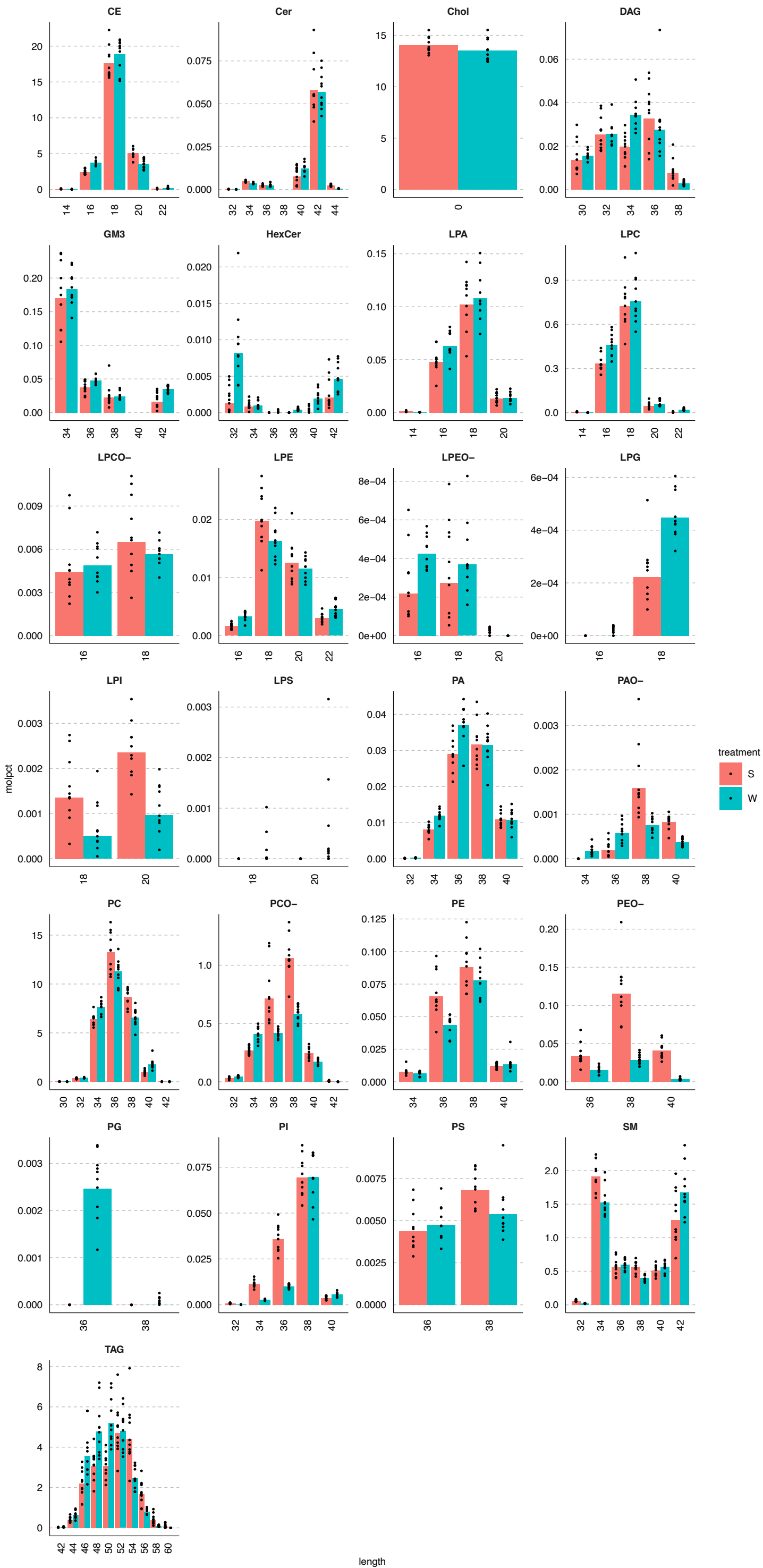

Supplement: S3 Fig — The y axes are shown in molar percentages (lipid quantities). The bars show the mean value in each of the two conditions “S” (summer active state) and “W” (winter hibernation). (PDF) [file pone.0291063.s005.pdf]

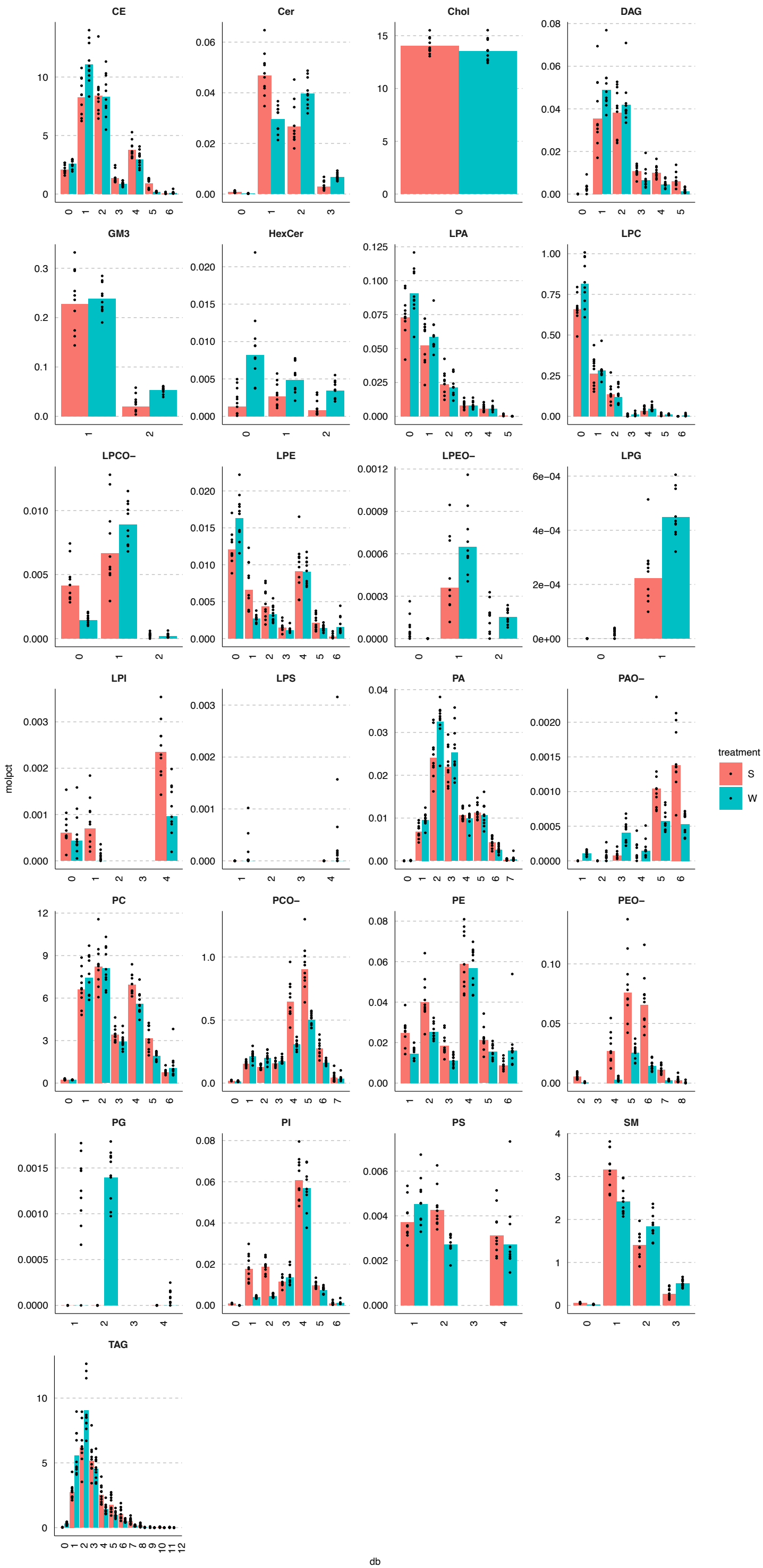

Supplement: S4 Fig — The y axes are shown in molar percentages (lipid quantities). The bars show the mean value in each of the two conditions “S” (summer active state) and “W” (winter hibernation). (PDF) [file pone.0291063.s006.pdf]

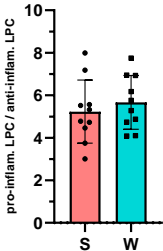

Supplement: S5 Fig — Pro-inflammatory lipids are classified as lipids with 0 or 1 double bonds, whereas anti-inflammatory lipids have 2–6 double bonds. A lower ratio indicates higher levels of anti-inflammatory LPCs and thus a lower proportion of inflammation. The ratio was calculated separately for each bear sample, and all samples were then pooled together in the two conditions. “S” is summer active state samples, “W” winter hibernation samples. A paired t test showed that no significant difference existed between the groups (p = 0.46). (PDF) [file pone.0291063.s007.pdf]
